# Propylthiouracil-Induced Vasculitis in Relapsed Graves’ Disease With Recent Iodine Exposure: A Therapeutic Dilemma

**DOI:** 10.7759/cureus.96700

**Published:** 2025-11-12

**Authors:** Sarmad Butt, MS Majeed, Iftikhar Ali, Mahnam Sattar, Umar Jawed

**Affiliations:** 1 Endocrinology and Diabetes, Ulster Hospital, Belfast, GBR; 2 Pharmacy Unit, Paraplegic Centre Peshawar, Peshawar, PAK; 3 Medicine, Jinnah Medical and Dental College, Karachi, PAK; 4 Internal Medicine, Northampton General Hospital, Northampton, GBR

**Keywords:** antineutrophil cytoplasmic antibodies, carbimazole, contrast induced iodine load, graves' disease, propylthiouracil, treatment dilemma, vasculitis

## Abstract

We present a challenging case of Graves’ disease complicated by propylthiouracil (PTU)-induced antineutrophil cytoplasmic antibody (ANCA)-positive vasculitis and recent iodine contrast exposure, creating a therapeutic dilemma where standard treatment options were severely limited. A 60-year-old man was diagnosed with Graves’ disease in late 2020 and treated with carbimazole for one year. When the medication was stopped, he relapsed and was switched to PTU due to side effects on restarting carbimazole. Although radioactive iodine (RAI) therapy was offered, the patient declined due to personal reasons. Nearly two years into PTU therapy, he developed worsening breathlessness, and computed tomography (CT) chest revealed a new pericardial effusion. He was treated presumptively for pericarditis, but follow-up imaging showed minimal improvement. Approximately four and a half years after the initial diagnosis, he experienced migratory joint pain, swelling, and persistently raised inflammatory markers. During his work-up, he received a significant iodine load from contrast-enhanced imaging. Autoimmune screening confirmed ANCA-associated vasculitis with anti-myeloperoxidase antibody positivity. PTU was discontinued, and corticosteroids were initiated, leading to clinical improvement. Given florid hyperthyroidism and recent iodine exposure, his case was reviewed at the Endocrine multidisciplinary meeting. Carbimazole was restarted as a bridging therapy; despite its vasculitis risk, it remained the best option until RAI, which was ineffective due to iodine saturation, could be given. Surgical thyroidectomy was unsafe due to severe thyrotoxicosis. These limitations created a narrow therapeutic window requiring careful multidisciplinary planning. This case highlights the rare but serious complication of PTU-induced vasculitis and the complexities of managing florid hyperthyroidism when drug intolerance, recent iodine exposure, and surgical risk converge, truly an endocrinologist’s worst nightmare.

## Introduction

Hyperthyroidism, most frequently caused by Graves’ disease, is a prevalent endocrine condition characterized by excessive thyroid hormone production and associated with significant global morbidity and mortality [1]. Antithyroid drugs (ATDs), including thionamides such as carbimazole (CBZ), methimazole, and propylthiouracil (PTU), are recommended and widely used as the initial modality; nonetheless, prolonged use is linked with unusual but potentially fatal adverse reactions [2,3]. PTU, in particular, has been associated with autoimmune sequelae, including drug-induced lupus and antineutrophil cytoplasmic antibody (ANCA)-associated vasculitis, which can involve pulmonary, renal, or systemic manifestations [4,5]. The incidence of PTU-induced vasculitis is low, occurring in less than 1% of treated patients, yet its clinical consequences can be severe, necessitating drug withdrawal and immunosuppressive therapy [4,6].

Exposure to iodinated contrast agents presents another therapeutic challenge in managing Graves’ disease, which can precipitate or exacerbate thyrotoxicosis and delay the efficacy of radioactive iodine (RAI) therapy [7]. This creates a narrow therapeutic window when patients exhibit intolerance or contraindications to both ATDs and definitive therapies such as RAI or thyroidectomy. Such scenarios demand a detailed, multidisciplinary approach to minimize morbidity and mortality while balancing the risks of drug-induced autoimmune complications and uncontrolled thyrotoxicosis.

We present a case of florid autoimmune hyperthyroidism in a patient with PTU-induced vasculitis and recent contrast iodine exposure, illustrating the complexities of therapeutic decision-making when standard treatment modalities are limited. This case not only emphasises the importance of vigilance for rare ATD-induced complications but also highlights the unique challenges faced by clinicians in managing severe hyperthyroidism under such circumstances.

## Case presentation

A 60-year-old man was diagnosed with Graves’ thyrotoxicosis following symptoms of hyperthyroidism. Laboratory investigations at presentation revealed suppressed thyroid-stimulating hormone, elevated free thyroxine, and positive thyroid receptor antibodies (TRAb), confirming the diagnosis. He achieved biochemical remission with CBZ 10 mg once a day (OD) after 12 months of therapy. However, on stopping treatment, he went into relapse. On re-initiation of CBZ, he developed peri-orbital swelling, prompting the patient to stop the medication. This side effect was mild and non-life-threatening. Based on the Naranjo scale, it scored 4, indicating a possible adverse drug reaction (ADR).

At around 21 months from initial diagnosis, he was initiated on PTU 50 mg twice daily, later titrated to 200 mg twice daily due to persistent hyperthyroidism. By 2.5 years after diagnosis, while on PTU 200 mg BD, his thyroid function tests (TFTs) demonstrated near-control. During this period, he declined RAI therapy despite repeated counselling, opting to defer definitive treatment due to personal reasons.

**Figure 1:**
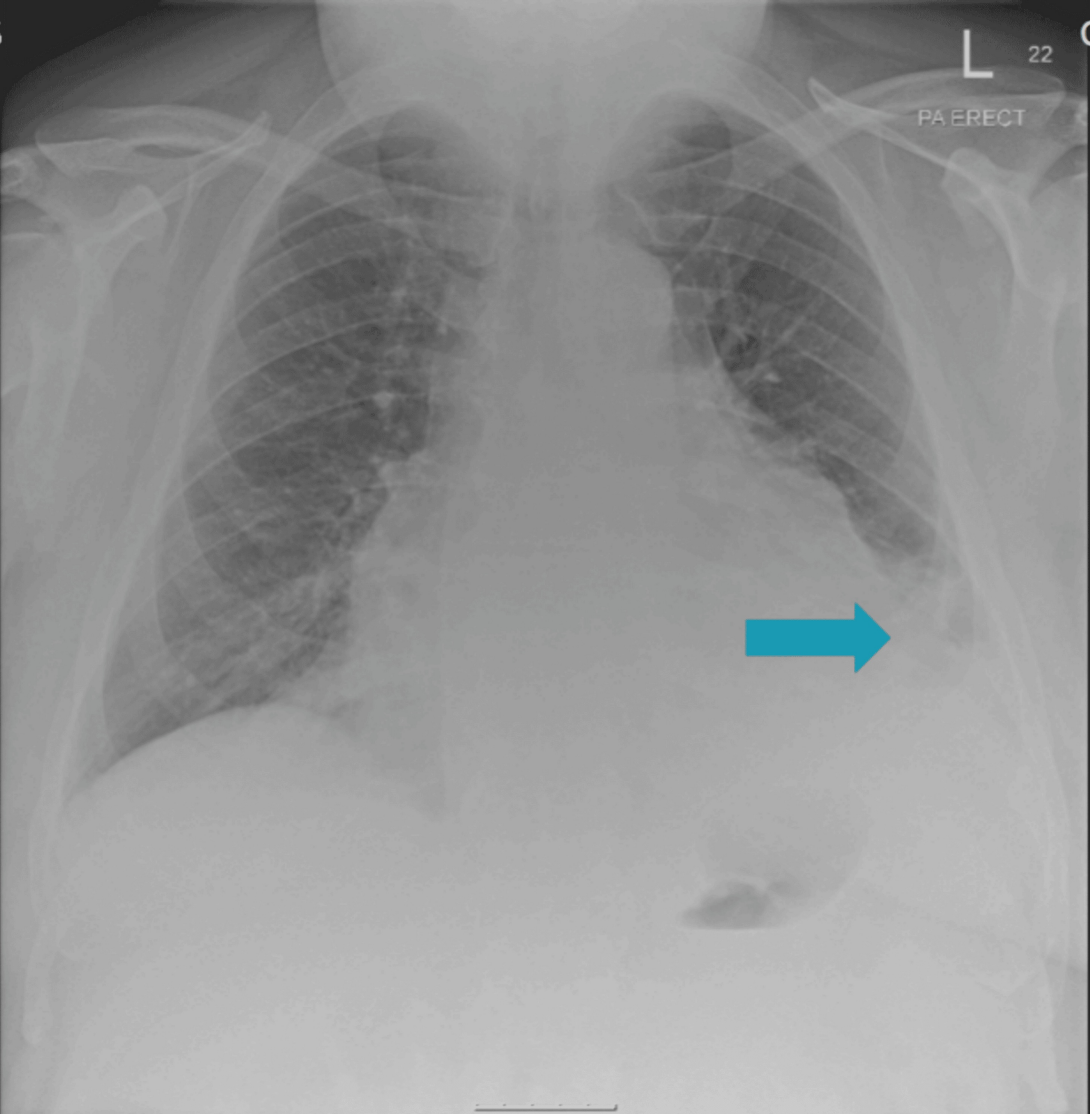
Postero-anterior chest radiograph demonstrating an enlarged cardiac silhouette with blunting of the left costophrenic angle, suggestive of a moderate pericardial effusion.

**Figure 2:**
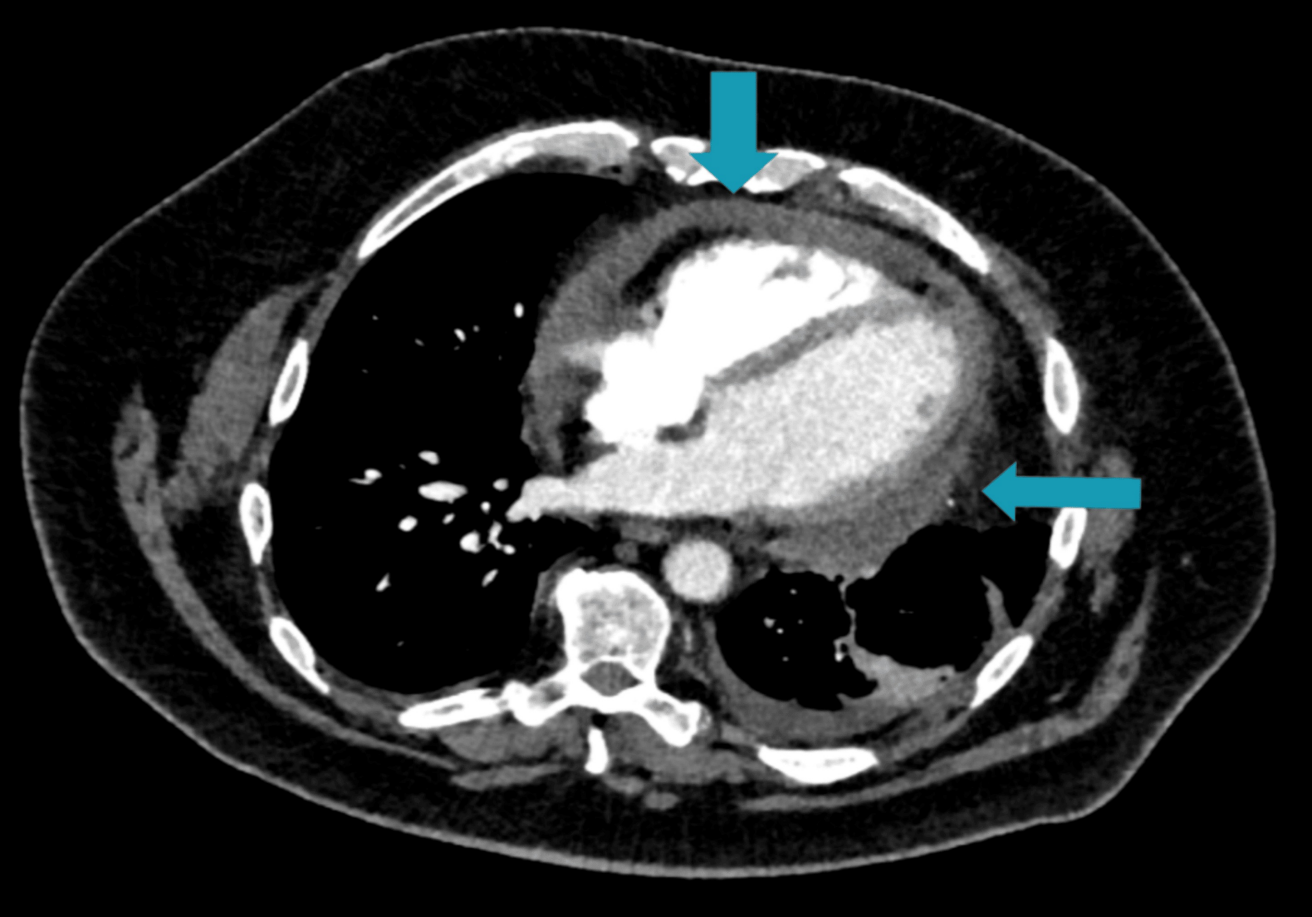
Contrast-enhanced axial CT chest demonstrating a moderate pericardial effusion (hypodense rim surrounding the heart) with associated inflammatory stranding in the adjacent pericardial fat and mediastinal lymphadenopathy, raising the possibility of pericarditis.

**Figure 3:**
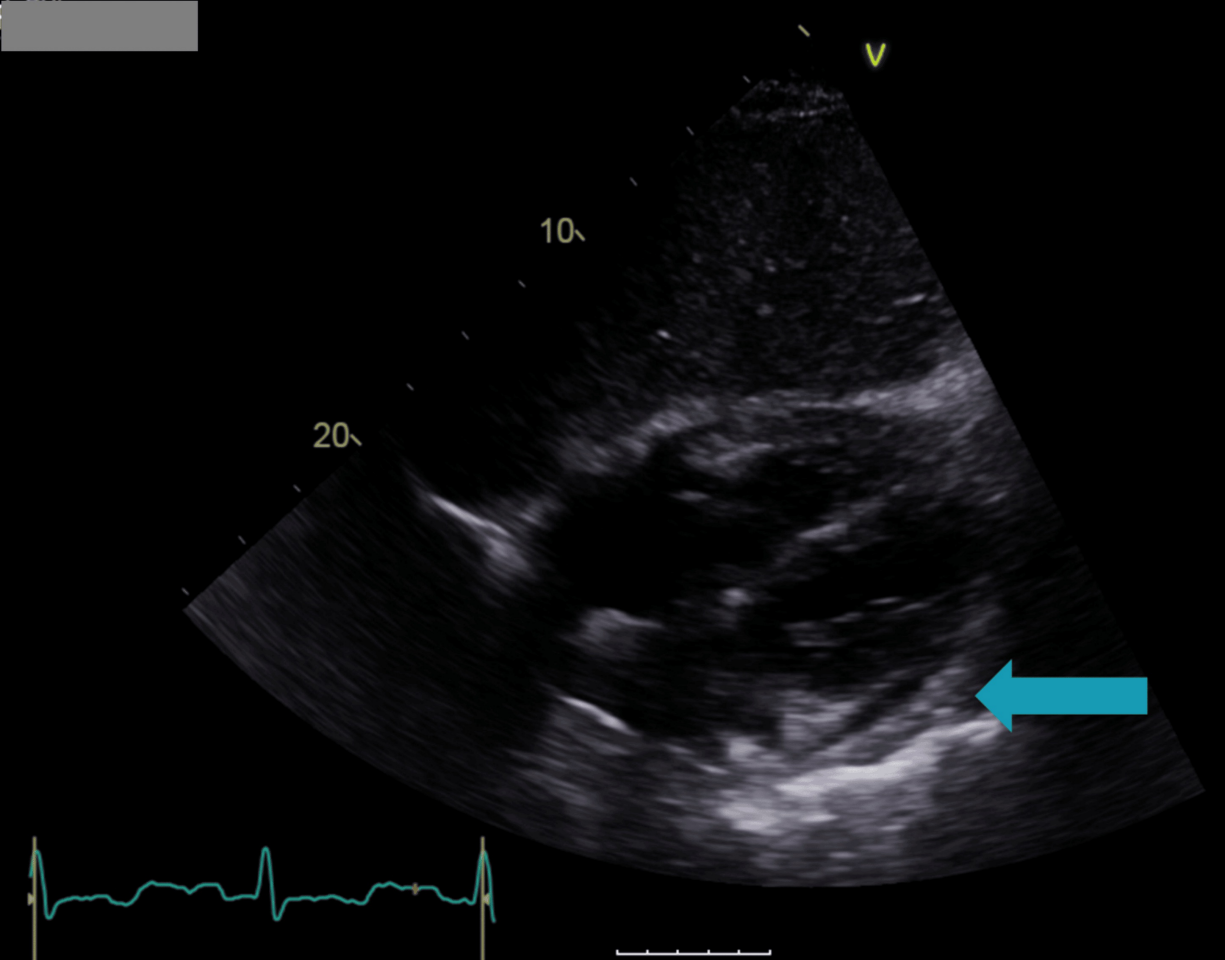
Echocardiographic parasternal long-axis view showing a circumferential anechoic space around the heart, consistent with pericardial effusion.

After about four years, the patient developed progressive exertional breathlessness, with multiple emergency presentations treated as presumed chest infections. A chest X-ray showed signs of left pleural effusion, which correlated with subsequent CT and echocardiographic findings (Figure 1). CT pulmonary angiography (CTPA) revealed a moderate pericardial effusion with inflammatory stranding of the adjacent pericardial fat and prominent mediastinal lymph nodes, raising suspicion of pericarditis (Figure 2), while echocardiography confirmed the effusion with preserved left ventricular function (Figure 3). He was treated with colchicine 500 micrograms twice daily for presumed pericarditis and followed up by cardiology; however, follow-up after three months demonstrated minimal improvement.

Approximately 4.5 years after diagnosis, he developed migratory arthralgia affecting the metacarpophalangeal joints, wrists, shoulders, and knees, with intermittent swelling. Rheumatology assessment revealed persistently raised inflammatory markers, strongly positive p-ANCA, elevated anti-myeloperoxidase (MPO) antibodies, and borderline raised proteinase 3 (PR3) antibodies. Urinalysis demonstrated 3+ blood and 2+ glucose. A diagnosis of PTU-induced ANCA-associated vasculitis was established, and PTU was discontinued after consultation with endocrinology. At this point, when PTU was discontinued, his TFTs were relatively stable. Based on the Naranjo scale, PTU scored 7, indicating a probable ADR (clear association with ANCA-associated vasculitis), establishing it as the primary culprit for the autoimmune complication. Oral corticosteroids (30 mg OD for seven days) were initiated, leading to rapid improvement in systemic and joint symptoms. This dose was tapered by 5mg weekly until a stable dose of 5 mg OD was achieved.

However, within days of PTU withdrawal, he presented to the ED with severe thyrotoxicosis, including symptoms of tremors and palpitations. Given the recent significant iodine load from contrast-enhanced CT imaging, RAI therapy was deemed ineffective in the short term, for at least eight weeks, and surgical thyroidectomy was considered but deemed unsafe due to uncontrolled thyrotoxicosis and cardiovascular risks. After multidisciplinary discussion, CBZ (10 mg OD) was cautiously reintroduced as a bridging therapy, with close monitoring for recurrence of adverse reactions despite his prior intolerance, with a plan for RAI therapy once iodine levels normalise.

Chronological details of investigations and events are given in Table 1.

**Table 1:** Chronological investigations and events leading up to the diagnosis of PTU-induced vasculitis Findings highlight the transition from initial hyperthyroidism to remission, subsequent relapse, development of vasculitis, and eventual florid thyrotoxicosis following PTU withdrawal. TSH: thyroid-stimulating hormone; FT4: free thyroxine; TRAb: thyrotropin receptor antibody; CTPA: CT pulmonary angiogram; p-ANCA: perinuclear anti-neutrophil cytoplasmic antibodies; MPO: myeloperoxidase; PR3: proteinase 3; ESR: erythrocyte sedimentation rate; CBZ: carbamazepine; PTU: propylthiouracil

Time from Diagnosis (months)	Investigations	Reference range (units)	Significant Findings/Interpretation	Management
0	TSH <0.05	0.27-4.20 (mIU/L)	Hyperthyroidism diagnosed	CBZ started
0	FT4 38.0	12-22 (pmol/L)		
0	TRAb 29.9	<1.75 (IU/L)		
12	TSH 1.55	0.27-4.20 (mIU/L)	Euthyroid - remission achieved	CBZ stopped
12	FT4 15.4	12-22 (pmol/L)		
19	TSH <0.05	0.27-4.20 (mIU/L)	Relapse	PTU started
19	FT4 24.3	12-22 (pmol/L)		
31	TSH 0.03	0.27-4.20 (mIU/L)	Partial control	PTU dose up titrated
31	FT4 12.6	12-22 (pmol/L)		
47	Chest X-ray		Left-sided unilateral effusion	CTPA performed
47	CTPA		Pericardial effusion	Referred to cardiology
47	Echocardiography		Pericardial effusion confirmed/pericarditis suspected.	Started on colchicine 500 micrograms
53	p-ANCA >1:320	Positive at >1:320	ANCA-associated vasculitis	PTU stopped
53	Urine dipstick: +blood, +glucose			
53	MPO 78.6	0.0-5.9 (IU/mL)		
53	PR3 21.1	0.0-4.9 (IU/mL)		
53	ESR 34	1-12 (mm/h)		
54	TSH <0.01	0.27-4.20 (mIU/L)	Florid thyrotoxicosis	CBZ restarted as bridging therapy while awaiting RAI after discussion in endocrine MDM
54	FT4>100	12-22 (pmol/L)		

The patient was successfully managed with corticosteroids for vasculitis, cessation of PTU, and cautious re-initiation of CBZ as interim therapy. He remains clinically stable on low-dose CBZ while awaiting definitive RAI therapy, with ongoing multidisciplinary follow-up involving endocrinology and rheumatology. 

## Discussion

This case report highlights the uncommon but fatal complication of PTU-induced ANCA-associated vasculitis in a male patient with relapsed Graves’ thyrotoxicosis. ATDs remain a cornerstone of initial management, yet both CBZ and PTU carry important safety considerations, as established in prior literature [2-6]. Our patient initially achieved remission with CBZ, but later developed peri-orbital swelling, necessitating a switch to PTU. Over the course of therapy, he developed systemic vasculitic features, including migratory arthritis and pericardial effusion, with laboratory evidence of high p-ANCA titres and MPO positivity, confirming PTU-induced ANCA-associated vasculitis.

Discontinuation of PTU, coupled with corticosteroid initiation, led to improvement in vasculitic manifestations. However, PTU withdrawal precipitated severe thyrotoxicosis, creating a therapeutic dilemma that required individualized, risk-balanced decision-making. 

From a pathophysiological standpoint, the temporal relationship between prolonged PTU exposure and the emergence of systemic inflammation strongly supports a drug-induced mechanism consistent with prior reports [4,6]. PTU has been shown to accumulate within neutrophils and alter myeloperoxidase configuration, promoting autoantibody formation in genetically susceptible individuals [8]. PTU is the antithyroid agent most commonly implicated in ANCA-positive vasculitis, particularly with anti-MPO antibodies [2,3,6]. In most reported cases, clinical manifestations range from mild cutaneous vasculitis to renal involvement [2-6,9,10], a pattern often described in the literature; however, our patient presented with an atypical cardiovascular manifestation (pericardial effusion). This expands the clinical spectrum of PTU-induced vasculitis and emphasizes the importance of considering drug toxicity as a differential in patients developing unexplained systemic symptoms on long-term thionamide therapy.

Management in this case diverged from standard recommendations for relapsed Graves’ disease. Guidelines typically advocate definitive treatment with either RAI or thyroidectomy in patients with antithyroid drug intolerance [11,12]. However, recent iodine exposure from contrast imaging rendered RAI temporarily ineffective, while ongoing systemic illness (uncontrolled thyrotoxicosis and vasculitis) and cardiovascular risk precluded safe thyroidectomy. In such settings, bridging therapy with ATDs may be unavoidable despite prior adverse events. The decision for cautious CBZ re-challenge was justified, given the mild nature of the initial reaction, its low Naranjo score (possible ADR), and the outcome of a multidisciplinary favorable benefit-risk reassessment. This approach, though unconventional, illustrates pragmatic management beyond guideline algorithms, where safety and disease control must be dynamically balanced. This case underscores the practical complexities that often extend beyond standard guideline recommendations.

The clinical trajectory of this patient demonstrates both consistencies and deviations from published data. Similar to prior reports, PTU was confirmed as the culprit agent, with laboratory findings (positive p-ANCA and MPO positivity) mirroring established immunological patterns of drug-induced vasculitis. Consistent with the literature, withdrawal of PTU led to symptomatic improvement when combined with weaning doses of corticosteroid therapy [3,9,10].

However, several aspects of this case diverge from typical patterns. While published cases frequently describe renal or dermatological involvement [5,10], our patient’s course was dominated by pericardial effusion and systemic inflammatory symptoms, highlighting an atypical but clinically significant presentation. These findings underscore the heterogeneous clinical spectrum of PTU-induced ANCA-associated vasculitis and the potential for multi-organ involvement if diagnosis is delayed. Moreover, literature often presents CBZ or methimazole intolerance as an absolute barrier to re-initiation [12]. In contrast, our case demonstrates that, under multidisciplinary supervision, cautious reintroduction of CBZ may serve as a bridging strategy when definitive therapy is temporarily contraindicated.

Several limitations in our case merit mention. The absence of baseline urine dipstick studies precludes assessment of possible renal involvement. A biopsy was not performed, limiting our ability to characterize the histopathologic nature or extent of the vasculitic process. Moreover, the precise interval for post-contrast iodine clearance was not documented, and serial urinary iodine monitoring, which could have better guided RAI timing, was unavailable. Despite these gaps, this case strengthens the established association between PTU and ANCA-associated vasculitis while introducing a novel cardiovascular phenotype and highlighting individualized management in complex endocrine emergencies.

Taken together, this case illustrates the dual challenge of balancing hyperthyroidism control while simultaneously managing life-threatening drug-induced vasculitis. Early clinical suspicion, prompt drug discontinuation, and a coordinated multidisciplinary approach with individualized therapeutic planning remain critical. The cautious reintroduction of CBZ as a bridging agent under close supervision provided an effective interim solution until definitive therapy could be safely undertaken.

## Conclusions

This case highlights the diagnostic and therapeutic challenges of managing florid Graves’ hyperthyroidism complicated by PTU-induced ANCA-associated vasculitis following recent iodine exposure. Conventional options were severely limited, as RAI therapy was temporarily ineffective, thyroidectomy was unsafe due to uncontrolled thyrotoxicosis, and both major ATDs carried potential risks. Multidisciplinary discussion enabled cautious reintroduction of CBZ as a bridging therapy, while corticosteroids successfully controlled vasculitis. This individualized, risk-balanced approach highlights the importance of timely definitive treatment planning and shared decision-making to optimize outcomes in complex clinical scenarios.

## References

[1] Wiersinga WM, Poppe KG, Effraimidis G (2023). Hyperthyroidism: aetiology, pathogenesis, diagnosis, management, complications, and prognosis. Lancet Diabetes Endocrinol.

[2] Kalbasi S, Tajik A, Ahmadi S, Khodabandeh H, Zare N, Bashirynejad M (2023). Propylthiouracil induced ANCA-positive vasculitis in a patient with Graves’ disease; a case report. J Parathyrd Dis.

[3] Khanolkar M, Owen P, Lazarus J (2004). Propylthiouracil induced ANCA positive vasculitis: a case report. Int J Endocrinol Metab.

[4] Paiaulla S, Venkategowda PM, Rao SM, Balaraju B (2015). Propylthiouracil-induced autoimmune disease. Indian J Crit Care Med.

[5] Galvis K, Sendos L (2024). #1707742 a case of PTU-induced vasculitis. Endocrine Practice.

[6] Kang DH, Song MK, Ju SH, Lee SI, Kang YE (2023). Propylthiouracil-induced antineutrophil cytoplasmic antibody-positive vasculitis and agranulocytosis: a rare case with life-threatening multiple systemic manifestations. Endocrinol Metab (Seoul).

[7] Dunne P, Kaimal N, MacDonald J, Syed AA (2013). Iodinated contrast-induced thyrotoxicosis. CMAJ.

[8] Gunton JE, Stiel J, Clifton-Bligh P, Wilmshurst E, McElduff A (2000). Prevalence of positive anti-neutrophil cytoplasmic antibody (ANCA) in patients receiving anti-thyroid medication. Eur J Endocrinol.

[9] Suárez Laurés AM, Quiñones L, Torres A, Pobes A, Forascepi R (2010). A case of p-ANCA-positive vasculitis with associated pericardial effusion [Article in Spanish]. Nefrologia.

[10] Almeida MS, Ramalho C, Gomes F, Ginga MD, Vilchez J (2022). Propylthiouracil-induced skin vasculitis. Cureus.

[11] Wu VT, Lorenzen AW, Beck AC (2017). Comparative analysis of radioactive iodine versus thyroidectomy for definitive treatment of Graves disease. Surgery.

[12] Ata F, Khan AA, Tahir S, Al Amer Z (2023). Carbimazole-resistant Graves' thyrotoxicosis is a diagnostic and therapeutic dilemma, case report with literature review. Int Med Case Rep J.

